# Etiology and Recovery of Neuromuscular Fatigue following Competitive Soccer Match-Play

**DOI:** 10.3389/fphys.2017.00831

**Published:** 2017-10-25

**Authors:** Callum G. Brownstein, Jack P. Dent, Paul Parker, Kirsty M. Hicks, Glyn Howatson, Stuart Goodall, Kevin Thomas

**Affiliations:** ^1^Department of Sport, Exercise and Rehabilitation, Faculty of Health and Life Sciences, Northumbria University, Newcastle-upon-Tyne, United Kingdom; ^2^Water Research Group, School of Environmental Sciences and Development, Northwest University, Potchefstroom, South Africa

**Keywords:** central, fatigue, peripheral, recovery, soccer, transcranial magnetic stimulation

## Abstract

**Aim:** Previous research into the etiology of neuromuscular fatigue following competitive soccer match-play has primarily focused on peripheral perturbations, with limited research assessing central nervous system function in the days post-match. The aim of the present study was to examine the contribution and time-course of recovery of central and peripheral factors toward neuromuscular fatigue following competitive soccer match-play.

**Methods:** Sixteen male semi-professional soccer players completed a 90-min soccer match. Pre-, post- and at 24, 48, and 72 h participants completed a battery of neuromuscular, physical, and perceptual tests. Maximal voluntary contraction force (MVC) and twitch responses to electrical (femoral nerve) and transcranial magnetic stimulation (TMS) of the motor cortex during isometric knee-extension and at rest were measured to assess central nervous system (voluntary activation, VA) and muscle contractile (potentiated twitch force, Q_tw, pot_) function. Electromyography responses of the *rectus femoris* to single- and paired-pulse TMS were used to assess corticospinal excitability and short-interval intracortical inhibition (SICI), respectively. Fatigue and perceptions of muscle soreness were assessed via visual analog scales, and physical function was assessed through measures of jump (countermovement jump height and reactive strength index) and sprint performance.

**Results:** Competitive match-play elicited significant post-match declines in MVC force (−14%, *P* < 0.001) that persisted for 48 h (−4%, *P* = 0.01), before recovering by 72 h post-exercise. VA (motor point stimulation) was reduced immediately post-match (−8%, *P* < 0.001), and remained depressed at 24 h (−5%, *P* = 0.01) before recovering by 48 h post-exercise. Q_tw,pot_ was reduced post-match (−14%, *P* < 0.001), remained depressed at 24 h (−6%, *P* = 0.01), before recovering by 48 h post-exercise. No changes were evident in corticospinal excitability or SICI. Jump performance took 48 h to recover, while perceptions of fatigue persisted at 72 h.

**Conclusion:** Competitive soccer match-play elicits substantial impairments in central nervous system and muscle function, requiring up to 48 h to resolve. The results of the study could have important implications for fixture scheduling, the optimal management of the training process, squad rotation during congested competitive schedules, and the implementation of appropriate recovery interventions.

## Introduction

Association football (soccer) is characterized by intermittent bouts of high-intensity activity interspersed with periods of low-to-moderate-intensity exercise (Mohr et al., 2003). During competitive match-play, players perform a diverse range of physically demanding actions, such as sprinting, jumping, accelerating, and changing direction, imposing significant disturbances on multiple physiological systems (de Hoyo et al., 2016). An inevitable consequence of these physical demands is fatigue, a sensation of tiredness and weakness during and following exercise, which is underpinned and/or modulated by a myriad of physiological and psychological processes. During match-play, fatigue manifests through transient reductions in work rate following the most demanding periods of a match and cumulative declines in work-rate during the latter stages of a match (Mohr et al., 2003; Rampinini et al., 2011). The fatigue induced by soccer match-play also persists post-exercise, and can take days to resolve. This notwithstanding, the competitive schedule in modern day soccer is such that teams are frequently required to play multiple games with minimal recovery periods between successive matches. As such, understanding the etiology of fatigue can provide important information for research and practice concerning recovery interventions aimed at alleviating fatigue and accelerating recovery.

The contributors to the fatigue experienced after soccer match-play have been previously related to energy depletion (Ekblom, 1986), perturbations to peripheral homeostasis (Ispirlidis et al., 2008), and damage to muscle tissue (Nédélec et al., 2012), which manifest in reductions in the force generating capacity of the quadriceps muscles (Thomas et al., 2017). Reductions in maximum voluntary contraction (MVC) strength, termed “muscle fatigue” (Gandevia, 2001), and performance during field tests of physical function (e.g., vertical jump and sprint tests) in response to match-play have been studied extensively (Rampinini et al., 2011; Nédélec et al., 2012; Thomas et al., 2017). Decrements in MVC post-exercise are typically attributed to neuromuscular fatigue; that is a consequence of impairments in contractile function (so-called “peripheral fatigue”), and/or the capacity of the central nervous system (CNS) to activate muscle (so-called “central fatigue”; Gandevia, 2001). Peripheral contributors to muscle fatigue can be assessed by measuring involuntary evoked responses to electrical stimulation at rest. Additionally, central contributors can be examined through evoked responses to electrical and magnetic stimulation during voluntary contractions, thereby permitting greater insight into the neuromuscular determinants of muscle fatigue following competitive soccer match-play. The physiological mechanisms that contribute to fatigue in the days post-soccer-match-play, however, have typically been studied from a peripheral viewpoint, with research focusing on disturbances at sites at or distal to the neuromuscular junction. In turn, the theoretical framework of recovery interventions in soccer has targeted events within the exercised muscle, such as exercise-induced muscle damage (EIMD) and substrate depletion (Nédélec et al., 2013).

While soccer match-play induces peripheral perturbations which contribute toward muscle fatigue (Nédélec et al., 2012), recent research has highlighted dissociated rates between the temporal pattern of recovery of muscle fatigue, and markers of EIMD following intermittent-sprint exercise (Pointon et al., 2012; Minett and Duffield, 2014). These findings have led to the suggestion that processes within the CNS could be contributing to the resolution of fatigue following soccer match-play (Minett and Duffield, 2014). In support of this suggestion, recent evidence demonstrated that impairments in CNS function (measured through reductions in voluntary activation, VA) were substantial following a simulated soccer match, and persisted for up to 72 h (Thomas et al., 2017). Similarly, following competitive soccer match-play, Rampinini et al. (2011) found a significant decline in VA which persisted for up to 48 h post-match. While these studies suggest that central factors are likely to contribute to post-match fatigue, research pertaining to recovery of CNS function following competitive match-play remains limited (Thomas et al., 2017), and recent reviews have highlighted the need for further work in this area (Minett and Duffield, 2014; Rattray et al., 2015).

The application of single- and paired-pulse transcranial magnetic stimulation (TMS) paradigms on fatigue, and the recovery of CNS function, following competitive match-play. Single-pulse TMS permits the quantitative assessment of corticospinal excitability through comparison of the motor evoked potential (MEP) amplitude with the maximum muscle compound action potential elicited by stimulation of the motor nerve (Sidhu et al., 2009). Paired-pulse TMS, in which a subthreshold conditioning stimulus precedes a suprathreshold test stimulus at a 2–5 ms interval, allows for the study of intracortical gamma-aminobutyric acid type A (GABA_a_) mediated inhibitory (short-interval intracortical inhibitory; SICI) circuits (Kujirai et al., 1993). These methods have previously been applied during single-limb isometric (Kennedy et al., 2016) and locomotor exercise (Sidhu et al., 2017) to reveal fatigue-induced changes in corticospinal excitability and/or SICI. As such, it is possible that changes in the status of these variables could be implicated in impaired CNS function following competitive soccer match-play. A recent study from our laboratory found no change in SICI and a reduction in corticospinal excitability 24 h following a simulated soccer match (Thomas et al., 2017). However, while soccer match simulations are designed to replicate the physiological demands of competitive match-play (Nicholas et al., 2000), many aspects of a real match aren't fully replicated through laboratory simulations (Magalhaes et al., 2010). For example, laboratory simulations do not include the perceptual demands associated with decision making, reacting and anticipating, and the mechanical and neuromuscular demands associated with the diverse range of physically demanding activities involved during match-play (Williams, 2000; Magalhaes et al., 2010). Given that changes in corticospinal excitability and SICI have been shown to be task specific (Maruyama et al., 2006; Sidhu et al., 2012), it is unclear whether differences in the demands of a simulated and competitive soccer match could influence the responses of these variables. Thus, the role of corticospinal excitability and SICI in post-exercise fatigue and recovery warrants further investigation.

Insight pertaining to the mechanisms of neuromuscular fatigue and the time-scale of recovery could have important practical implications in regards to the optimal management of the training and recovery process, player rotation strategies during congested competitive schedules, and for those involved in the scheduling of matches. The primary aim of the present study was to examine the contribution and time-course of recovery of peripheral and central factors toward neuromuscular fatigue following competitive soccer match-play. Furthermore, while practitioners regularly implement physical and perceptual measures in order to assess readiness to train/compete, the sensitivity of these measures to neuromuscular fatigue is unclear. As such, a secondary aim of the study was to assess the relationship between the temporal pattern of recovery of neuromuscular fatigue and a range of physical and perceptual measures following match-play in order to provide practitioners with simple tools to monitor the physical and cognitive contributors to fatigue in the days post-soccer-match-play. We hypothesized that competitive soccer match-play would elicit substantial neuromuscular fatigue from both central and peripheral origin which would persist in the days post-exercise, with concurrent decrements in measures of physical performance.

## Materials and methods

### Participants

After receiving ethical approval from the Northumbria University Faculty of Health and Life Sciences Ethics committee in accordance with the ethical standards established in the Declaration of Helsinki, 16 male semi-professional Association Football players (six defenders, seven midfielders, and three forwards; age: 21 ± 1 years; stature: 1.77 ± 0.06 m; body mass: 78 ± 8 kg) from Level 9 of the English Football League gave written informed consent to participate in the study. Players trained three to four times a week in addition to at least one competitive match. Participants were required to refrain from physical activity and alcohol consumption for the duration of the study and in the 48-h prior to data collection, and abstain from caffeine consumption for the 12-h prior to each experimental visit. The study was conducted 1 week after the completion of the competitive season while players were still accustomed to their normal training and game load.

### Design

A practice visit preceded the main trial for habituation to the measurement tools employed in the study. For the experimental trial, participants were required to attend the laboratory on 4 consecutive days, separated by 24 h at the same time of day. On the first day, participants completed a 90-min competitive soccer match (two 45 min halves with a 15 min rest interval). Pre-, post- and on subsequent days at 24, 48, and 72 h post-exercise, participants completed assessments of neuromuscular, physical, and perceptual function to ascertain the time-course of recovery of these variables following competitive soccer match-play (see supplementary digital content 1, schematic of experimental protocol).

### Procedures

#### Practice trial

Prior to the data collection, participants attended a practice visit during which they were habituated with the measurement tools and study protocol. The procedures used during the main trial were explained, before participants performed a practice trial consisting of the neuromuscular, physical and perceptual measures employed in the study (described below).

### Experimental trials

#### Competitive soccer match

On the day of the first experimental trial, participants attended the laboratory for baseline measurements (described in detail below). Subsequently, the players completed a 90 min competitive soccer match consisting of two 45 min halves interspersed by a 15 min recovery interval. The study took place across two games separated by 1 week, with eight players investigated following game one and eight following game two. Both games took place on an outdoor synthetic pitch at the same time of day (13:00), with ambient temperature of 15 and 18°C and air humidity of 57 and 72% for games one and two, respectively. The games consisted of 22 players (2 goalkeepers and 20 outfield players) and five substitutes, with players assigned to one of two different teams, of the same level, competing against each other. Players retained their normal playing positions during the games. The 16 participants being investigated participated in the full match and were not substituted. In order to ensure the match was competitive in nature and to create the physical and psychological environment of a normal competition, coaches and managers were present at both games and provided verbal encouragement throughout. The games were refereed by officials from the Northumberland Football Association, and were registered as official matches under the English Football Association. During the game, players were allowed to drink water *ad libitum*. The activity profiles and heart rates of the players were measured throughout the games using global positioning system (GPS) with built in heart rate monitors (Polar Team Pro, Polar Electro Oy, Finland).

#### Outcome measures

A range of neuromuscular, physical and perceptual measures were assessed pre- and post-match, and at 24, 48, and 72 h post-match. Details of these measures are provided below. In order to ensure a timely capture of data during the post-match assessment, an abridged version of the testing protocol was administered to measure central nervous system and muscle function, consisting of measures of VA (using motor nerve and motor cortical stimulation) and potentiated quadriceps twitch force (Q_tw,pot_) (see supplementary digital content 1, schematic of experimental protocol). In addition, a “conveyer belt” system was applied to the perceptual, neuromuscular and physical measurements, in which one player finished one set of tests, and the subsequent player began testing. Overall, the post-match assessment took place between 10 and 60 min following the match. Details of these measures are provided below.

#### Perceptual responses

Participants completed the “*Elite Performance Readiness Questionnaire”* (Dean et al., 1990), a measure of performance readiness consisting of 10 subjective measures of fatigue, soreness, motivation to train, anger, confusion, depression, tension, alertness, confidence, and sleep. Participants drew a vertical line on a 100 mm horizontal line in response to questions used for each measure, such as “how fatigued do you feel?,” “how sore do your muscles feel?,” and “how motivated to train do you feel?” Each scale was anchored with verbal descriptors “not at all” to “extremely.” Perceptual measures were assessed at each time-point prior to commencing the warm-up. In addition, participants completed a “readiness to train post warm-up” analog scale immediately after the warm-up at pre-, 24, 48, and 72 h post.

#### Assessment of neuromuscular function

The evoked quadriceps force and electromyographic (EMG) responses of the *rectus femoris* to TMS of the primary motor cortex, and electrical stimulation of the femoral nerve, were used to assess the contribution of central and peripheral mechanisms toward neuromuscular fatigue and recovery in the quadriceps. The quadriceps were studied as this muscle group incurs significant decrements in function as a result of soccer match-play (Rampinini et al., 2011). A calibrated load cell (MuscleLab force sensor 300, Ergotest technology, Norway) recorded muscle force (N) during an isometric maximal voluntary contraction (MVC) of the knee extensors. Muscle fatigue was inferred from reductions in MVC force. During contractions, participants sat with hips and knees at 90° flexion, with a load cell fixed to a custom-built chair and attached to the participants right leg, superior to the ankle malleoli, with a noncompliant cuff. Electrical activity from the *rectus femoris* (RF) and *bicep femoris* (BF) were recorded from surface electrodes (Ag/AgCl; Kendall H87PG/F, Covidien, Mansfield, MA, USA) placed 2 cm apart over the belly of each muscle, with a reference electrode placed on the patella. Electrode placement was marked with indelible ink to ensure consistent placement throughout the study, with the areas cleaned and shaved prior to electrode placement. The electrodes recorded electrical activity in the RF and BF, with the signal processed to permit analysis of the root-mean-square (RMS) amplitude for sub-maximal and maximal voluntary contractions, the compound muscle action potential (M-wave) from the electrical stimulation of the femoral nerve, and the motor evoked potential (MEP) elicited by TMS. Signals were amplified: gain ×1,000 for EMG and ×300 for force (CED 1902; Cambridge Electronic Design, Cambridge, UK), band-pass filtered (EMG only: 20–200 Hz), digitized (4 kHz; CED 1401, Cambridge Electronic Design) and analyzed offline. Further details on these methods are provided below.

#### Motor nerve stimulation

Motor nerve stimulation was used for the measurement of contractile function, muscle membrane excitability and estimated VA. Single and paired electrical stimuli (100 Hz) were administered using square wave pulses (200 μs) via a constant-current stimulator (DS7AH, Digitimer Ltd., Hertfordshire, UK) using self-adhesive surface electrodes (CF3200, Nidd Valley Medical Ltd., North Yorkshire, UK). Electrical stimuli was first administered to the motor nerve at rest in 20 mA step-wise increments from 20 mA until the maximum quadriceps twitch amplitude (Q_tw_, N) and muscle compound action potential (M_max_, mV) were elicited. To ensure a consistent, supramaximal stimulus and account for any activity-induced changes in axonal excitability, the resulting stimulation intensity was increased by 30% (189 ± 35 mA). The peak-to-peak amplitude and area of the electrically evoked maximal compound action potential (M_max_) was used as a measure of membrane excitability. Participants subsequently completed six isometric MVCs (3–5 s duration) of the knee extensors, separated by 60 s rest. For the final three MVCs, paired electrical stimuli (100 Hz) were delivered during and 2 s post contraction to assess VA. Single pulse electrical stimuli were delivered 5 s post-MVC to assess potentiated quadriceps twitch force (Q_tw,pot_). In addition, the following mechanical measures of muscle contractility were derived from the single pulse potentiated twitch response: contraction time (CT, time to peak twitch tension), maximum rate of force development (MRFD, maximal value of the first derivative of the force signal), maximal rate of relaxation (MRR, lowest value of the first derivate of the force signal), and one half relaxation time (R.T_0.5_, time taken for twitch force to decay to half of the peak twitch amplitude; Goodall et al., 2015).

#### Motor cortical stimulation

Single- and paired-pulse TMS of 1 ms duration were delivered using a concave double cone coil using two linked monopulse magnetic stimulators (Magstim 200, The Magstim Company Ltd, Whitland, UK). The junction of the double cone coil was aligned tangentially to the sagittal plane, with its center 1–2 cm to the left of the vertex. The optimal coil placement was determined at the start of each trial as the position that elicited the largest MEP in the RF, with a concomitant small MEP in the BF. The position was marked with indelible ink on the scalp for consistent placement during subsequent trials. The stimulator intensity was based on active motor threshold (AMT) measured during a 10% MVC (Thomas et al., 2017). In order to determine AMT, the stimulator intensity was increased in 5% steps beginning at 35% of stimulator output until a consistent MEP with peak-to-peak amplitudes of >200 μV was found. Thereafter, stimulus intensity was reduced in 1% step until an MEP of >200 μV was found in 50% of stimulations (mean stimulation intensity, 37 ± 5%).

#### Motor evoked potential recruitment curve (stimulus-response curve)

Once AMT was established, the stimulator intensities required to assess the MEP response to varying TMS intensities were determined. The recruitment curve (or stimulus-response curve) was constructed by delivering TMS at a range of intensities relative to AMT, and assessing average MEP amplitude at each intensity (Carson et al., 2013). Participants held a light voluntary contraction (10% MVC) with one set of five stimuli delivered at each of 90, 100, 110, 120, 130, 140, 150, and 160% of AMT in a randomized and counterbalanced order, with 4–6 s between each stimuli and 15 s between each set.

#### Short-interval intracortical inhibition (SICI)

Ten single and 10 paired-pulse TMS stimuli were administered in two sets of 10 stimuli during a 10% MVC, for measurement of unconditioned and conditioned MEP amplitude, respectively. Paired-pulse TMS consisted of a subthreshold conditioning pulse at 70% of AMT, and a suprathreshold test pulse at 120% AMT, with an inter-stimulus interval (ISI) of 2 ms. Single- and paired-pulses were delivered in a pre-determined randomized order, with 4–6 s between each stimulation and a short rest between each set. The ratio of conditioned to unconditioned MEP amplitude was used as a measure of SICI.

#### Voluntary activation with TMS

Single pulse TMS was delivered during brief (3–5 s) contractions at 100, 75, and 50% MVC, separated by 5 s of rest, for determination of voluntary activation with TMS (VA_TMS_). This procedure was repeated 3 times with 15 s rest between each set. The stimulation intensity (68 ± 8%) was set at the stimulator output that elicited the maximum superimposed twitch force during a 50% MVC (Thomas et al., 2017).

#### Assessment of physical function

Participants completed a battery of assessments to measure physical function in variables relevant to optimal football performance. All measures of physical function were performed following the neuromuscular assessment and the completion of a standardized warm-up. Jump height (cm) during a countermovement jump (CMJ) and reactive strength index (RSI) during a drop jump (DJ) were measured using an optical timing system (Optojump Next, Microgate, Milan, Italy). For CMJ, participants started from an erect position with hands akimbo. On verbal command, participants made a downward countermovement before jumping vertically for maximum height. For reactive strength index (DJ-RSI), participants were instructed to step off a 30 cm box with hands akimbo, before jumping vertically for maximum height as soon as possible after landing. To ensure the DJ-RSI was assessing fast stretch-shortening cycle, a maximum ground contact time of 200 ms was allowed during each jump, with participants given visual feedback on each ground contact time and jump height after each jump. Reactive strength index (cm·s^−1^) was calculated as the ratio between jump height (cm) and ground contract time (s). All participants were given three attempts at each jump with 60 s between each repetition. Linear speed (20 m sprint with 10 m splits) during three maximal effort sprints was recorded using electronic timing gates (TC Timing Systems, Brower Timing Systems, Draper, USA). Sprints were self-initiated from a standing start 30 cm behind the first timing gate, with participants encouraged to sprint maximally through the timing gate at 20 m.

#### Match play physical performance and intensity

During the games, GPS with built in HR monitors (Polar Team Pro, Polar Electro Oy, Finland) were used to assess total distance (TD), high-intensity running (HIR, distance covered at running velocities higher than 15 km·h), total accelerations (>1 m·s^−2^), total decelerations (>-1 m·s^−2^), and mean and peak HR (Akenhead et al., 2013). These variables were compared between games, and with the season averages recorded during competitive matches in the sample group to ensure the matches elicited a physical demand comparable to that experienced during normal competition.

#### Creatine kinase

Fingertip samples of capillary blood were obtained at each time point and immediately assayed for creatine kinase (CK) concentration (Reflotron, Roche Diagnostics, Germany).

### Data analysis

Voluntary activation assessed through the interpolated twitch technique (Merton, 1954) was quantified by comparing the amplitude of the superimposed twitch force (SIT) to the potentiated twitch (100 Hz) delivered 2 s following the MVC at rest using the following equation: Motor point VA (%) = [1 − (SIT/Q_tw,pot_) × 100]. Voluntary activation using TMS was assessed during contractions at 50, 75, and 100% MVC using linear regression of the superimposed twitch force evoked by TMS (Thomas et al., 2017), with the regression analysis confirming a linear relationship at each time-point (*r*^2^ range = 0.89 ± 0.06–0.92 ± 0.04). The estimated resting twitch (ERT) was calculated as the *y*-intercept of the linear regression between the mean amplitude of the SIT force evoked by TMS at each contraction intensity. Subsequently, VA_TMS_ was quantified using the equation [1 − (SIT/ERT) × 100]. To quantify SICI, the ratio of the average conditioned paired-pulse MEP was expressed relative to the average unconditioned MEP at 120% AMT. Recruitment curves were constructed by plotting the TMS stimulation intensity relative to AMT against the MEP amplitude averaged from the five stimulations at each intensity, expressed relative to M_max_. The ratio of the MEP amplitude to the maximum M-wave was used as an index of corticospinal excitability. In order to provide a summary measure of corticospinal excitability, the summated area under the recruitment curve was calculated for each participant at each time point using the trapezoid integration method (Carson et al., 2013). The root mean square EMG amplitude (RMS_EMG_) and average force was calculated in the 80 ms prior to each TMS to ensure a similar level of background muscle activity was present during the recruitment curve and SICI measurements. Previous work from our laboratory has demonstrated moderate-to-high reproducibility of MEP amplitude [within-day ICC = 0.91, typical error (TE) expressed as a coefficient of variation (CV) = 17.7%, between-day ICC = 0.87, TE expressed as a CV = 25.3%] and SICI (within-day ICC = 0.84, TE expressed as a CV = 16.4%, between-day ICC = 0.74, TE expressed as a CV = 18.3%) and high reproducibility for pre-stimulation force (within-day ICC = 0.98, TE expressed as a CV = 4.4%, between-day ICC = 0.98, TE expressed as a CV = 4.1%) (unpublished observation). The peak-to-peak amplitude of evoked MEP and M_max_ were measured offline.

### Statistical analysis

Data are presented as mean ± *SD*. Repeated measures ANOVA was used to assess changes in each outcome measure over time (pre-, post-, 24, 48, and 72 h). Normality of the data was assessed using the Shapiro–Wilks test. Assumptions of sphericity were explored and controlled for all variables using the Greenhouse-Geisser adjustment, where necessary. In the event of a significant main effect, Dunnett's multiple comparison procedure was employed with the pre-trial score used as the control category. The assumptions underpinning these statistical procedures were verified as per the guidelines outlined by Newell et al. (2010). Paired sample *t*-tests were used to assess differences in match-running variables between the first and second halves of the competitive matches, as well the match-running data from the study and normative data gathered throughout the season. Independent sample *t*-tests were used to assess differences between the match demands of the two competitive matches in the study. Pearson product-moment correlations coefficients were calculated to determine relationships between pre-post changes in MVC, contractile (Q_tw,pot_) or CNS function (motor point and VA measured with) and match-running variables, as well as the association between the proximity of the post-match assessment to the end of the match and the pre-post changes in these variables. Spearman's rank-order correlation was used to assess the relationship between the temporal pattern of recovery of neuromuscular variables (Q_tw,pot_, motor point and VA measured with TMS), physical function tests (CMJ, DJ-RSI, and 20 m sprint) and perceptual responses (fatigue and soreness). For each variable, recovery was defined as a return of the respective value to within the measurement error relative to baseline derived from the baseline assessment and previous work (Rampinini et al., 2011). The relationship between the time-point (post-, 24, 48, 72 or >72 h post-match) at which neuromuscular fatigue, physical function, or perceptual responses recovered was then assessed. Standardized effect sizes (Cohen's *d*) were calculated for focused pairwise comparisons and interpreted as small (≥0.2), moderate (≥0.6), and large (≥1.2). All data were analyzed using Statistical Package for Social Sciences (SPSS version 22.0). Statistical significance was accepted at *P* < 0.05.

## Results

### Perceptual responses

Perceptual responses from the Elite Performance Readiness Questionnaire can be viewed in Table 1. Fatigue was higher than pre-match at post-, 24 and 48 h (all *P* < 0.001) and at 72 h (*P* = 0.001). Muscle soreness was higher than pre-match values at post-match, 24 and 48 h (*P* < 0.001) before recovering at 72 h (*P* = 0.16). Motivation to train was reduced at all-time points post-match (*P* < 0.001 at post-match and 24 h, *P* = 0.001 at 48 h and *P* = 0.01 at 72 h), while post-warm-up readiness to train was reduced at 24 h (*P* = 0.02) before recovering by 48 h (*P* = 0.16).

**Table 1:** Perceptual responses measured via visual analog scales (mm) pre-, post-, and 24, 48, and 72 h post- competitive soccer match-play (*n* = 16).

	**Pre-**	**Post-**	**24 h**	**48 h**	**72 h**
Fatigue	1.09 ± 0.97	7.31 ± 1.68^***^	6.17 ± 1.60^***^	4.18 ± 1.66^***^	2.60 ± 1.67^**^
Soreness	1.72 ± 1.67	7.05 ± 1.60^***^	6.48 ± 1.73^***^	5.03 ± 1.56^***^	2.33 ± 1.32
Motivated to train	6.89 ± 1.76	2.99 ± 2.58^***^	5.16 ± 2.45^***^	5.44 ± 2.16^**^	6.06 ± 1.84^*^
Anger	0.63 ± 0.86	1.45 ± 1.67	0.51 ± 0.64	1.24 ± 2.05	0.54 ± 0.67
Confusion	0.51 ± 0.69	0.75 ± 0.79	0.45 ± 0.55	0.44 ± 0.47	0.56 ± 0.62
Depression	0.34 ± 0.44	0.72 ± 1.46	0.44 ± 0.56	0.45 ± 0.52	0.53 ± 0.69
Tension	1.00 ± 1.13	3.84 ± 3.18^**^	2.53 ± 2.29^**^	2.76 ± 1.97^***^	1.74 ± 1.48
Alertness	6.25 ± 2.39	4.56 ± 2.65^*^	5.78 ± 2.30	5.61 ± 1.89	5.71 ± 2.29
Confidence	6.99 ± 1.84	6.89 ± 2.72	6.95 ± 2.39	6.98 ± 2.05	7.27 ± 2.16
Sleep	5.83 ± 2.02	N/A	6.77 ± 2.22	5.73 ± 1.63	5.79 ± 2.48
Post warm-up readiness to train	7.69 ± 1.97	N/A	5.17 ± 3.19^*^	6.76 ± 2.69	6.98 ± 2.99

Values are mean ± SD. Significant differences in comparison with baseline indicated by

* *p < 0.05*,

** p < 0.01 and

*** *p < 0.001*.

### Neuromuscular fatigue

**Figure 1:**
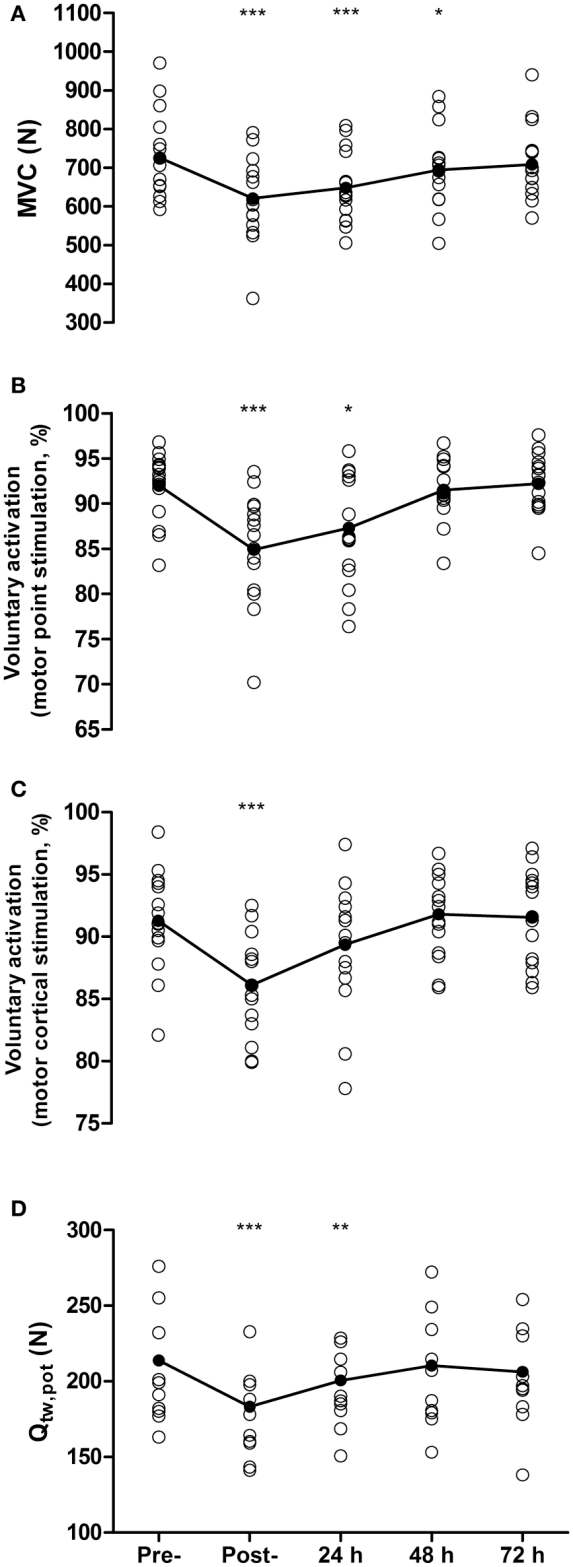
Maximal voluntary contraction force (MVC**, A**), voluntary activation measured with femoral nerve stimulation **(B)**, voluntary activation measured using motor cortical stimulation **(C)**, and quadriceps potentiated twitch force **(**Q_tw,pot,_**D)** measured pre-, post- and at 24, 48, and 72 h post- competitive soccer match-play (*n* = 16). Significant differences in comparison with baseline indicated by ^*^P ≤ 0.05, ^**^P ≤ 0.01, ^***^P ≤ 0.001. Individual responses are plotted, with lines representing the mean scores.

Maximal voluntary contraction force was reduced by 14 ± 9% from pre- to post-match (726 ± 109 N vs. 621 ± 106 N, *P* < 0.001, *d* = 0.89, Figure 1A), remained depressed at 48 h (695 ± 100 N, *P* = 0.01, *d* = 0.31), but recovered by 72 h (709 ± 94 N, *P* = 0.27, *d* = 0.17). Voluntary activation measured with motor nerve stimulation decreased by 7.1% from pre- to post-match (92.0 ± 3.7 vs. 84.9 ± 6%, *P* < 0.001, *d* = 1.16), remained depressed at 24 h by 4.7% (87.3 ± 6.0%, *P* = 0.01, *d* = 0.86), but recovered by 48 h (91.5 ± 3.1%, *P* = 0.61, *d* = 0.12, Figure 1B). Voluntary activation measured with motor cortical stimulation was reduced by 5.3% from pre- to post-match (91.4 ± 3.7 vs. 86.1 ± 4.03%, *P* < 0.001, *d* = 1.09), but had recovered by 24 h (89.3 ± 5.3%, *P* = 0.08, *d* = 0.41, Figure 1C). Potentiated twitch force was reduced by 14 ± 6% from pre- to post-match (214 ± 45 N vs. 183 ± 37 N, *P* < 0.001, *d* = 0.71), remained depressed by 6 ± 6% at 24 h (201 ± 37 N, *P* = 0.01, *d* = 0.32), but recovered by 48 h (210 ± 41 N, *P* = 0.19, *d* = 0.08, Figure 1D). The pre- to post-match decline in Q_tw,pot_ was accompanied by changes in peripherally derived measures of muscle contractility. Specifically, MRFD and CT were both reduced by 10 ± 15% (*P* = 0.008) and 8 ± 11% (*P* = 0.006), respectively, with both recovering by 24 h (*P* > 0.65). Maximum M-wave and RMS/M_max_ did not differ from baseline values at any time point (all *P* > 1.00). No significant correlation was found between the proximity of the post-match neuromuscular assessment to the end of the match and the magnitude of change in MVC (*r* = 0.24, *P* = 0.36), Q_tw,pot_ (*r* = 0.21, *P* = 0.43), VA measured with motor nerve (*r* = −0.18, *P* = 0.52), and motor cortical stimulation (*r* = 0.18, *P* = 0.51).

### Central nervous system excitability and inhibition

**Figure 2:**
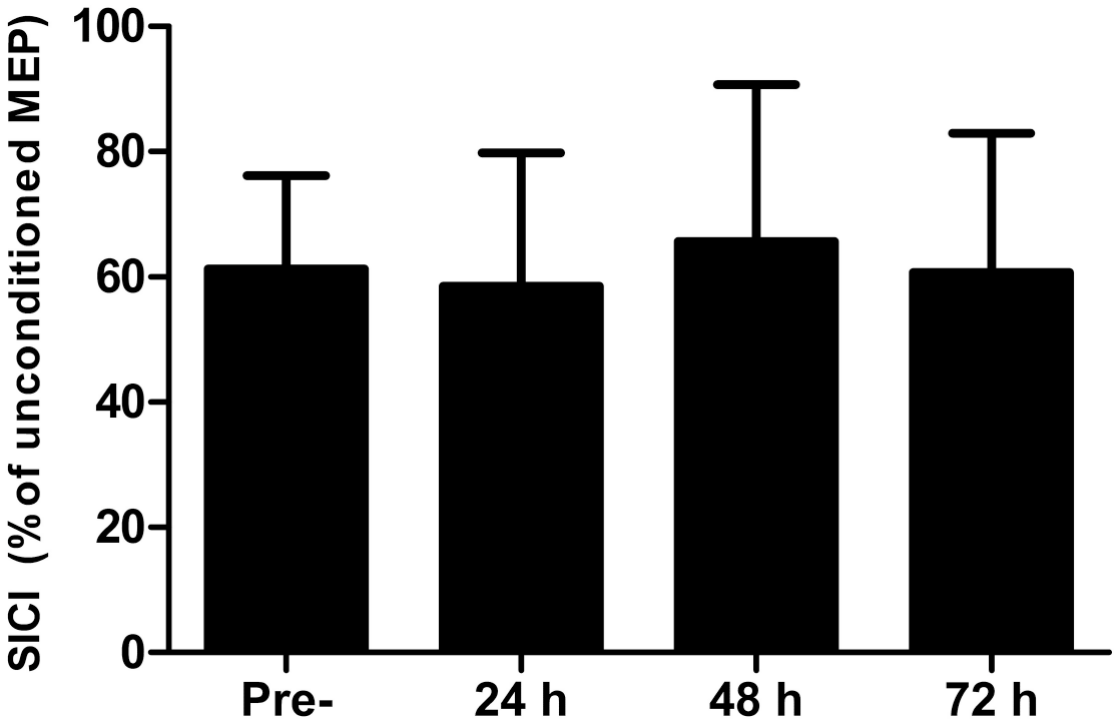
Short-interval intracortical inhibition (SICI) measured in the *rectus femoris* pre-, 24, 48, and 72 h post- competitive soccer match-play (*n* = 16). Values are mean + *SD*.

**Figure 3:**
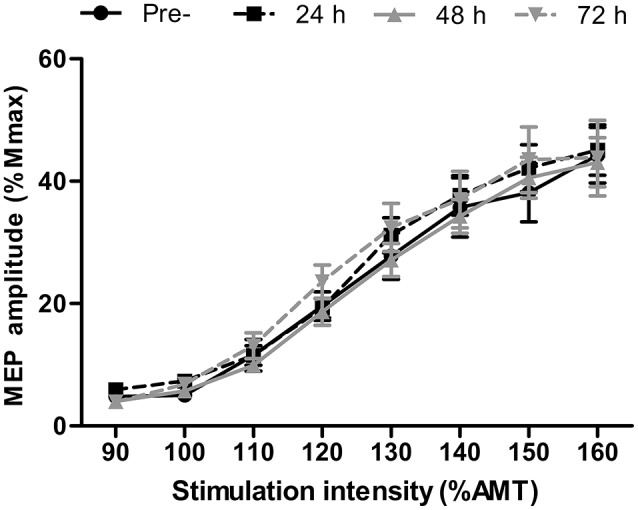
Recruitment curve displaying motor evoked potential (MEP) amplitude relative to the maximal compound muscle action potential (M_max_) in the *rectus femoris* at stimulation intensities relative to active motor threshold (AMT) at pre-, 24, 48, and 72 h post- competitive soccer match-play (*n* = 16). Values are mean ± *SD*.

There were no differences in AMT at any time-point (*P* > 0.13). Short-interval intracortical inhibition was not different to the baseline value at any time point (Figure 2; all *P* > 0.26). Similarly, corticospinal excitability, as inferred through the area under the recruitment curve, was not different at any time point (*P* > 0.65; Figure 3).

### Physical function

**Figure 4:**
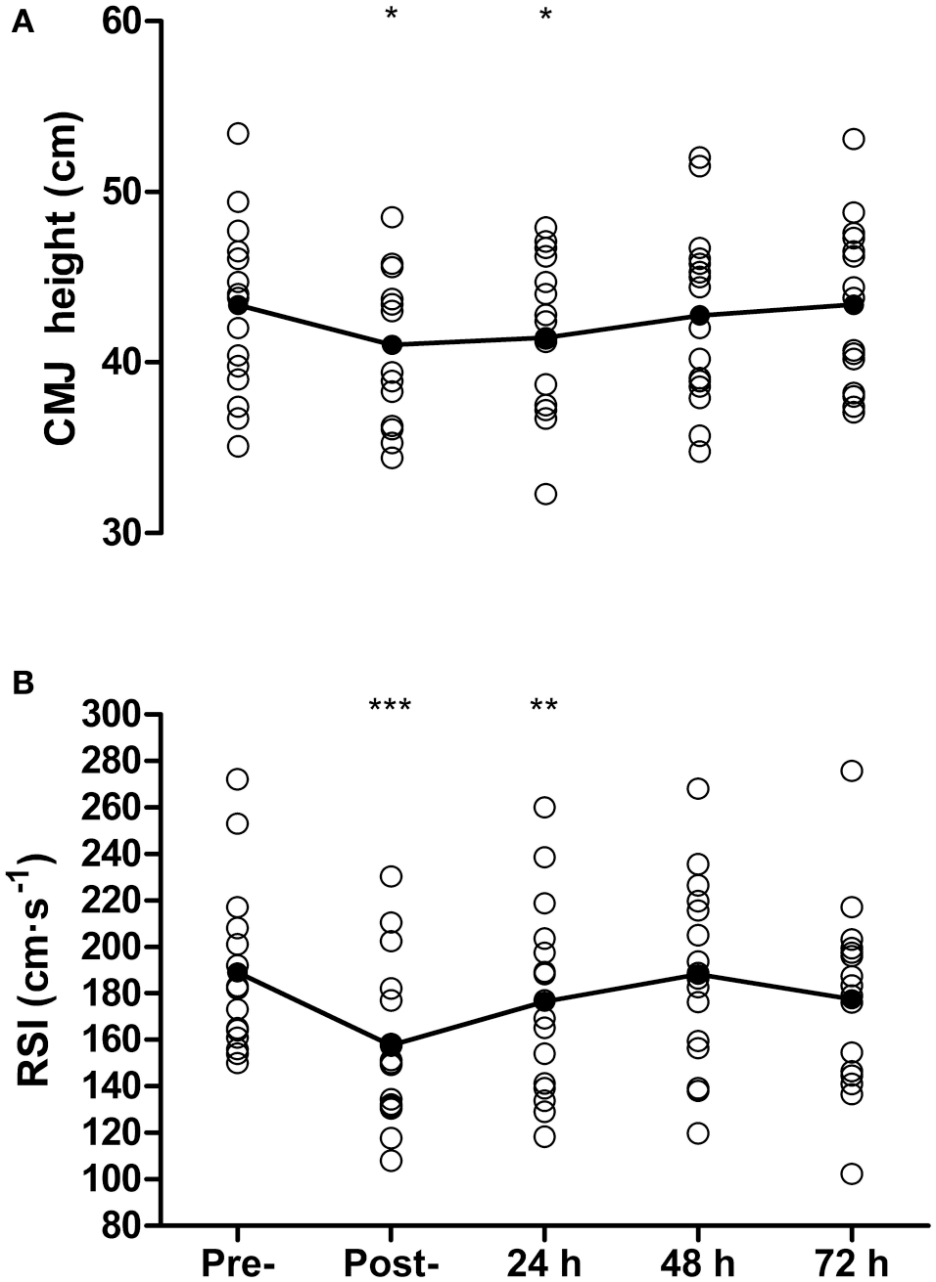
Countermovement jump height **(**CMJ, **A)**, and reactive strength index **(**RSI, **B**) measured pre-, post-, and 24, 48, and 72 h post- competitive soccer match-play (*n* = 16). Significant differences in comparison with baseline indicated by ^*^P ≤ 0.05, ^**^P ≤ 0.01, ^***^P ≤ 0.001. Individual responses are plotted, with lines representing the mean scores.

Countermovement jump height was reduced from pre- to post-match by 5 ± 8% (43.4 ± 5.1 vs. 41.0 ± 4.6 cm, *P* = 0.03, *d* = 0.47) and at 24 h by 4 ± 6% (41.5 ± 4.6 cm, *P* = 0.02, *d* = 0.39) but recovered by 48 h (42.8 ± 5.2 cm, *P* = 0.34, Figure 4A). For DJ measurements, contact time was 169 ± 16 ms at baseline, and was successfully maintained on subsequent days (range 169–173 ms). A reduction in RSI was found from pre- to post-match by 17 ± 8% (189 ± 35 vs. 158 ± 35 cm·s^−1^, *P* < 0.001, *d* = 0.83) that remained below baseline at 24 h post by 7 ± 9% (176 ± 41 cm·s^−1^, *P* = 0.01, *d* = 0.33), but recovered by 48 h (188 ± 40 cm·s^−1^, *P* = 0.88, Figure 4B). Sprinting performance over 20 m was reduced from pre- to post-match by 4 ± 2% (3.11 ± 0.11 vs. 3.23 ± 0.11 s, *P* < 0.001, *d* = 0.93) but recovered thereafter (all *P* > 0.45), while 10 m sprint time showed no decrease between baseline and any other time-point (all *P* > 0.07).

### Relationship between recovery of neuromuscular variables and physical and perceptual measures

Table 2 displays the relationships between the temporal pattern of recovery of neuromuscular fatigue and physical and perceptual measures. A significant correlation was found between recovery of motor point VA and CMJ (*r* = 0.831, *P* < 0.001), with no other significant correlations found with any other physical function or perceptual variable (all *P* > 0.07).

**Table 2:** Spearman's rank-order correlation coefficients between the temporal pattern of neuromuscular fatigue indicators and physical function and perceptual measures.

	**Q**_**tw, pot**_		**Motor point VA**		**VA measured with TMS**	
	***R*^2^**	***P***	***R*^2^**	***P***	***R*^2^**	***P***
CMJ	0.31	0.59	0.83^***^	< 0.01	−0.125	0.66
DJ-RSI	0.41	0.15	−0.08	0.78	0.141	0.62
10 m sprint	−0.28	0.29	0.34	0.19	−0.166	0.56
20 m sprint	−0.24	0.41	−0.10	0.71	0.439	0.10
Fatigue	0.46	0.07	0.08	0.77	−0.151	0.56
Soreness	−0.12	0.66	0.34	0.19	0.063	0.88

Significant correlation indicated by

*** *p < 0.001. Q_tw,pot_, quadriceps potentiated twitch force; VA, voluntary activation; TMS, transcranial magnetic stimulation; CMJ, countermovement jump; DJ-RSI, drop-jump RSI*.

### Match performance and intensity

Match activity and heart rate variables are presented in Table 3. In both games, reductions in total distance (game 1: −26 ± 18%, *P* < 0.001; game 2: −14 ± 6%, *P* = 0.01) and HIR (game 1: −22 ± 19%, *P* < 0.001; game 2: −35 ± 21%, *P* < 0.001) were found between the first and second halves. No differences were found in time-motion or heart rate variables between games 1 and 2, or with data gathered throughout the competitive season (all *P* > 0.11). No correlations were found between any of the match-running variables and pre-post changes in measures of neuromuscular function (all *P* > 0.16).

**Table 3:** Match activity and heart rate variables during competitive soccer match-play.

	**Total distance**	**High-intensity**	**Accels**	**Decels**	**Mean HR**	**Max HR**
	**(m)**	**(m)**	**(no.)**	**(no.)**	**(bpm)**	**(bpm)**
Pooled data	10,041 ± 626	1,211 ± 257	315 ± 64	208 ± 56	164 ± 11	193 ± 10
Game 1	10,037 ± 552	1,286 ± 199	301 ± 64	197 ± 49	170 ± 11	197 ± 12
Game 2	10,046 ± 770	1,126 ± 303	329 ± 64	218 ± 64	158 ± 7	189 ± 5
Season average	10,076 ± 1,363	1,456 ± 143	289 ± 97	204 ± 63	158 ± 12	194 ± 12

*The study data was gathered across two competitive matches, while normative data from the same players was gathered throughout the competitive season (n = 16). Values are mean ± SD. HIR, high-intensity running; Accels, accelerations; Decels, decelerations, HR, heart rate*.

### Creatine kinase

Creatine kinase (IU·L^−1^) increased from pre- to post-match (344 ± 319 vs. 872 ± 423 IU·L^−1^, *P* < 0.001), peaked at 24 h post (1,059 ± 571 IU·L^−1^, *P* < 0.001), and remained elevated at 48 h (763 ± 477 IU·L^−1^, *P* = 0.001) and 72 h post (537 ± 349 IU·L^−1^, *P* = 0.02).

## Discussion

The aim of this study was to examine the contribution and time-course of recovery of central and peripheral factors toward neuromuscular fatigue following competitive soccer match-play. The data indicate that competitive match-play elicits considerable neuromuscular fatigue that requires 48–72 h to resolve. Impairments in both VA and Q_tw,pot_ were substantial post-match, and persisted for up to 48 h following exercise, while reductions in the voluntary force generating capabilities of the knee extensors took 72 h to recover. A secondary aim of the study was to ascertain the relationship between the temporal pattern of recovery of neuromuscular fatigue and physical and perceptual measures following match-play in order to provide practitioners with simple tools to monitor fatigue and recovery. While a significant correlation was found between recovery of motor point VA and CMJ, no other significant associations were found, suggesting that the physical and perceptual measures used in the present study cannot be used as surrogate measures of neuromuscular fatigue in the quadriceps. Collectively, these data add to growing evidence that prolonged impairments in the capacity of the CNS to activate the quadriceps muscles are implicated in fatigue following intermittent sprint exercise (Rampinini et al., 2011; Thomas et al., 2017), and could have important implications for the optimization of the training process and the implementation of appropriate recovery interventions.

### Neuromuscular fatigue following competitive match-play

Following competitive soccer match-play, the players in the present study exhibited substantial reductions in VA and Q_tw,pot_. While previous investigations concerning the etiology of fatigue following match-play have predominantly focused on peripheral perturbations (Mohr et al., 2003; Ispirlidis et al., 2008; Nédélec et al., 2012), the results from the present study suggest that competitive match-play elicits impairments in CNS function which take days to resolve. Specifically, VA measured through motor point stimulation was significantly reduced post-match (7.1%) and remained depressed at 24 h (4.7%), before recovering by 48 h, while VA_TMS_ was reduced post-match (5.3%) before recovering by 24 h. The magnitude of impairments and time-course of recovery of VA was similar to that reported elsewhere following both competitive (Rampinini et al., 2011) and simulated match-play (Thomas et al., 2017). Accordingly, these results suggest that soccer match-play elicits prolonged impairments in the capacity of the CNS to activate the quadriceps muscles. While the functional relevance of this activation deficit cannot be accurately quantified, the results support recent suggestions that future research should place more emphasis on the recovery of CNS function following intermittent sprint exercise (Minett et al., 2014; Thomas et al., 2017).

While the precise mechanisms underpinning the prolonged impairments in the capacity of the CNS to activate muscle following long-duration locomotor exercise are unknown (Carroll et al., 2017), a number of potential factors might have contributed toward the residual activation deficit which persisted at 24 h. Group III and IV muscle afferents, which provide inhibitory feedback to various sites within the CNS (Sidhu et al., 2017), are sensitive to various markers of muscle injury, such as the release of biochemical substrates (e.g., bradykinin, histamines, and prostaglandins) and factors associated with inflammation (Endoh et al., 2005; Sidhu et al., 2009; Pitman and Semmler, 2012). Furthermore, there is some indication that elevations in the concentration of brain cytokines following eccentric exercise might also modulate recovery of CNS impairment (Carmichael et al., 2006; Goodall et al., 2016). Given that the repeated eccentric contractions associated with match-play are likely to have induced muscle damage, as evidenced by the increase in CK in the days post-exercise, and a subsequent inflammatory response (Ispirlidis et al., 2008), it is possible that the inflammatory response which ensues following match-play could have contributed to the residual activation deficit which persisted for up to 48 h post-match.

In addition to impairments in VA, substantial impairments in contractile function, assessed via Q_tw,pot_, were evident post-match and persisted at 24 h before recovering by 48 h. A decrease in the force output of the muscle in response to electrical stimulation can be attributed to metabolic and mechanical factors that negatively influence the excitation-contraction coupling process, as well as impairments in neuromuscular transmission at the sarcolemma (Allen et al., 2008). The lack of change in M_max_, a measure of neuromuscular transmission, combined with the significant reductions in peripherally derived measures of contractility (CT and MRFD), suggest that the contractile impairments demonstrated post-match were a result of disruptions occurring beyond the sarcolemma. Furthermore, many of the metabolic mechanisms thought to interfere with the excitation-contraction process, such as the accumulation of intramuscular metabolites (e.g., P_i_ and H^+^) and the depletion of cellular ATP levels, recover rapidly following exercise (Allen et al., 2008) and had likely returned close to pre-exercise levels by the time of the post-match assessment. The more prolonged reductions in Q_tw,pot_ evident in the present study were more likely the consequence of the large mechanical stress imposed on muscle fibers during match-play, which can lead to myofibrillar damage, disorganization of sarcomeres and interference with cellular Ca^2+^ handling (Skurvydas et al., 2016). This supposition is further supported through comparisons with previous data on recovery of Q_tw,pot_ following cycling exercise, which imposes large metabolic but little mechanical stress on the muscles and consequently leads to a more hastened recovery of contractile function. For example, following a 5 km cycling time-trial, Blain et al. (2016) found that Q_tw,pot_ had recovered 5 h post-exercise despite the immediate post-exercise reduction (~30%) being substantially higher than the present study. Thus, it is likely that the prolonged reduction in Q_tw,pot_ was primarily a result of mechanical damage incurred during match-play.

Maximum voluntary contraction (MVC) force remained below baseline at 48 h, despite there being no statistically significant difference in VA or Q_tw,pot_ between baseline and 48 h. Although, not statistically significant, both Q_tw,pot_ and motor point VA remained 1.8 and 0.5% below baseline 48 h post-match, respectively, with 7 of the 16 participants displaying a reduction in VA or Q_tw,pot_ at 48 h above the measurement error obtained from previous work from our laboratory (2.2–3.1% VA, 4.8–5.3% Q_tw,pot_; Goodall et al., 2017; Thomas et al., 2017). It is possible that these small decrements combined might explain the reduced MVC at 48 h. The MVC force loss at 48 h could also be explained by neuromuscular fatigue that was not fully captured by measures of VA and Q_tw,pot_, or a contribution of other physiological or psychological factors that could contribute to muscle fatigue, such as substrate depletion, inflammation or perceptions of muscle soreness. Furthermore, although MVC remained below baseline at 48 h, reductions were small, with absolute decrements equating to −32 ± 43 N, or −4 ± 6%; a value similar to the measurement error of this variable in our lab (4.0 and 4.4%; Thomas et al., 2015, 2016). As such, the functional relevance and meaningfulness of such small impairments could be questioned.

The magnitude of post-match neuromuscular fatigue in the present study was similar to that of previous work from our laboratory using a simulated soccer match protocol (Thomas et al., 2017). However, the time-course of recovery in the days post- was markedly faster in the present study. While MVC force had recovered by 72 h in the present study, small impairments in MVC persisted at this time-point after a simulated match (Thomas et al., 2017). Furthermore, although the post-match reduction in Q_tw,pot_ was similar to that observed after simulated match-play, the time-course of recovery of contractile function was substantially faster in the present study. Specifically, at 24 h following simulated soccer, recovery of Q_tw,pot_ was negligible (−14 ± 10% post-match to −13 ± 5% at 24 h) and remained below baseline at 72 h (Thomas et al., 2017). In contrast, recovery of Q_tw,pot_ at 24 h in the present study after a competitive match was substantial (−14 ± 6% post-match to −6 ± 6% at 24 h), and returned to baseline by 48 h. Two integral differences between the studies might explain the disparity between the results. Namely, the study by Thomas et al. (2017) was primarily conducted during the late off-season and early pre-season phase, while the current study was conducted a week following the competitive season, when players were conceivably in better physical condition and more accustomed to the demands of soccer match-play. The more rapid recovery of Q_tw,pot_ might reflect a mechanical adaptation of skeletal muscle acquired throughout the competitive season, acting to provide greater protection against the muscle damage sustained during match-play (Hoffman et al., 2005; Silva et al., 2014). Differences between match-related fatigue and the time course of recovery during different phases of training throughout the competitive season presents an interesting area for future investigation. In addition, Thomas et al. (2017) employed a simulated-match protocol with forced decelerations which, in contrast to the self-paced nature of competitive match-play, requires players to match running speeds with externally controlled stimuli. Although, competitive match-play includes an array of actions associated with eccentric contractions which subsequently lead to muscle damage, it is possible that players were less accustomed to the specific demands of the simulated match-protocol, resulting in greater muscle damage than during a competitive match, possibly contributing toward the slower time-course of recovery of muscle function and fatigue. Thus, caution should be exercised when making comparisons between simulations and competitive soccer matches, or using the two protocols interchangeably.

### Corticospinal excitability and short-intracortical inhibition

No changes were found in corticospinal excitability or SICI at any time-point throughout the study. We implemented a recruitment curve (or stimulus-response curve), which measures MEP amplitude normalized to the maximal M-wave in response to varying stimulation intensities relative to AMT, and has been suggested as the most sensitive measure of motor system excitability (Carson et al., 2013). However, no differences were found in the summated area under the recruitment curve, suggesting that corticospinal excitability does not change in the days following competitive football match-play. Furthermore, while changes in MEP amplitude in response to fatigue might depend on the level of force at TMS delivery (Gruet et al., 2013), no changes were found in MEP amplitude elicited during measurement of cortical VA at 50, 75, or 100% MVC, further indicating a lack of change in corticospinal excitability. The excitability of corticospinal cells to fatiguing exercise seems to be task specific, with several studies reporting altered corticospinal excitability in response to fatiguing isometric exercise in isolated upper (Maruyama et al., 2006) and lower limb (Mileva et al., 2012) models, whereas numerous studies report no change in response to various modes of locomotor exercise (Sidhu et al., 2012; Goodall et al., 2015). Discrepancies between studies involving isometric and locomotor exercise can likely be explained by differences in the systemic and local responses between the two types of exercise, which might differentially influence the responsiveness of corticospinal cells (Sidhu et al., 2012). The lack of change in corticospinal excitability in the present study is thus consistent with previous findings following locomotor exercise (Sidhu et al., 2012; Goodall et al., 2015). Similarly, no changes were found in SICI, which reflects intracortical inhibition mediated by GABA_a_, following competitive match-play, corroborating the findings of Thomas et al. (2017). Previous studies which have found changes in SICI in response to locomotor (Sidhu et al., 2012) and isometric exercise (Hunter et al., 2016) have noted that exercise-induced changes in the excitability of inhibitory circuits are short lasting, and dissipate within minutes of exercise cessation. Thus, the lack of change in SICI post-exercise might not fully reflect modulations in SICI that could occur during exercise. While it is possible that any change in SICI and corticospinal excitability would have resolved by the time measurements were taken, the lack of change in these measures in the days post-match suggests that this measure plays a negligible role in the residual perturbations in CNS function which occurs following competitive match-play.

### Recovery of physical function

Jump performance (CMJ and drop jump for RSI) was significantly impaired post-match and at 24 h, before recovering by 48 h. The time-course of recovery in jump performance is similar to that reported following competitive match-play (Ispirlidis et al., 2008; Nédélec et al., 2012). In contrast to the CMJ and drop jump, 20 m sprint performance was impaired post-match but recovered thereafter. The superior sensitivity of CMJ and drop jumps to altered neuromuscular function compared with 20 m sprint time has been reported elsewhere (Gathercole et al., 2015; Thomas et al., 2017). Although, the temporal pattern of recovery of vertical jump performance and neuromuscular fatigue was similar on a group level, correlation analysis showed only one significant relationship between recovery of motor point VA and CMJ height, with no other significant associations between any of the neuromuscular and physical or perceptual measures. A number of possible explanations could account for the discrepancies between recovery of neuromuscular fatigue and physical and perceptual measures. Namely, there is an inherent level of variability associated with measures of voluntary performance, with individuals able to alter their jump or sprint mechanics in an attempt to maximize performance (Ratel et al., 2006). For example, it has previously been suggested that individuals alter their jump mechanics when fatigued in order to help maintain jump height (Gathercole et al., 2015). In addition, measures of neuromuscular function target the knee extensors under isometric conditions, while jump performance involves multi-joint dynamic movements, which could further contribute to the discrepancies. Thus, the divergence in the temporal pattern of recovery suggests that using the physical function and perceptual measures employed in the present study as surrogate measures of neuromuscular fatigue in the quadriceps is inappropriate.

### Recovery of perceptual responses

Competitive match-play resulted in fatigue, perceptions of soreness and tension, and decreases in alertness and motivation to train in the hours and days post-exercise. Despite neuromuscular function and physical performance measures having returned to baseline, differences in fatigue and motivation to train persisted at 72 h post-exercise. A lack of association between subjective and objective indicators of fatigue has previously been reported (Saw et al., 2016), and provides support for the inclusion of both when monitoring recovery following match-play to provide a more comprehensive understanding of an athlete's physical and psychological readiness to train/compete. Although, neuromuscular function and physical performance measures had returned to baseline by 72 h, it is possible that the inflammatory response, which ensues following match-play (Ispirlidis et al., 2008) and exacerbates fatigue (Smith, 2000), persisted at 72 h, potentially explaining the differences in recovery of perceptual and neuromuscular responses. The divergent recovery of fatigue compared to measures of neuromuscular function emphasizes the aforementioned multi-factorial nature of fatigue elicited by the varied mechanical, metabolic and cognitive demands of soccer match-play. A possible alternative explanation for the self-reported fatigue which persisted at 72 h is that a bias effect might exist during measurements of perceptual responses, in which participants feel inclined to report elevated levels of fatigue relative to baseline at each post-match assessment. Similar to the findings of Thomas et al. (2017), perceptions of readiness to train assessed after a standardized warm-up recovered faster than that assessed at rest. Specifically, readiness to train returned to baseline by 48 h when assessed post-warm up and remained below baseline at 72 h when measured at rest. This provides further support for the suggestion that a warm-up could mask perceptions of fatigue in the days post-match (Thomas et al., 2017), and practitioners should consider the timing of perceptual assessments when monitoring fatigue following match-play.

### Match performance and intensity

The performance indices and physiological demands of the competitive match intervention were similar to data gathered from the same team throughout the competitive season, and not different between matches. Total running distance and mean and peak HR were comparable to values reported in elite level professional footballers (Ekblom, 1986; Mohr et al., 2003). High-intensity running distance, which has previously been shown to be a distinguishing factor between top-class players and those at a lower level (Mohr et al., 2003), was similar to that described for players of the same level (O'Donoghue et al., 2001), and lower than values reported in elite players (Mohr et al., 2003; Rampinini et al., 2011). The significant decline in TD and HIR between the first and second halves indicates that players were experiencing match-related fatigue during the fixtures, suggesting that the physical demands imposed on the players were similar to those during real competition.

### Limitations and future directions

The demands of competitive football match-play are inherently unpredictable, and a high degree of inter-individual variability in match activity exists. Consequently, the magnitude of fatigue and in turn, the time-course of recovery, can be highly variable between subjects. Thus, although competitive match-play is the most valid model of investigating the mechanisms of fatigue and time-scale of recovery, one limitation of this method compared with a laboratory simulation is the lack of experimental control over the activity profiles of the players and the high inter-subject variability in match demands. In order to account for this limitation, the relationships between match-running variables and the changes in measures of neuromuscular function from pre-to-post match were assessed, with no significant associations found. Overall, the results of the present study provide valuable information on the fatigue and recovery following a real match; an ecologically valid stimulus that includes movements, skill and cognitive demands that aren't fully replicated by a laboratory simulation. Moreover, it should be noted that measurements of neuromuscular fatigue were performed in the quadriceps muscles only. Other muscle groups of the lower extremity, such as the hamstrings, calves and hip adductors/abductors, also play an important role in many match-related actions and likely incur decrements in function following match-play. However, measuring neuromuscular fatigue in many of these muscles groups is fraught with difficulties, such as targeting a specific muscle group through motor nerve and/or cortical stimulation, and participant discomfort associated with receiving motor nerve stimulation. Additionally, the quadriceps muscles are heavily involved in actions associated with football match-play, and have previously been reported to incur significant fatigue as a result of match-play (Rampinini et al., 2011; Thomas et al., 2017), thus making them a suitable choice when assessing match-related neuromuscular fatigue. Due to the logistical constraints of taking multiple measures on a large group of participants in a short time frame, neuromuscular and physical assessments took place from 10 to 60 min post-match. In this time, some aspects of neuromuscular fatigue might have dissipated by the time of measurement, while it could also be argued that those who had neuromuscular measures taken at a closer proximity to the end of the match might have been more fatigued than those assessed later. However, correlation analysis showed no significant relationship between the proximity of the post-match neuromuscular assessment to the end of the match and the magnitude of muscle fatigue. Furthermore, the level of post-match reductions in CNS and contractile function were similar to or higher than that found in previous studies (Rampinini et al., 2011; Thomas et al., 2017), demonstrating the robust nature of the data.

## Conclusions

Competitive soccer match-play induced impairments in both VA and Q_tw,pot_, requiring up to 48 h to return to baseline, while reductions in MVC took 72 h to recover. While previous research on post-match fatigue and recovery has predominantly focused on the recovery of contractile function, the results of the present study suggest that competitive match-play elicits substantial deficits in CNS function, requiring up to 48 h to resolve. Decrements in contractile function showed a similar time-course of recovery, and can likely be attributed to disturbances in excitation-contraction coupling as a result of the muscle damage incurred during match-play. While measures of vertical jump performance followed a comparable time-course of recovery to neuromuscular fatigue on a group-level, only one significant association was found between recovery of neuromuscular fatigue and measures of physical function or perceptual responses.

## Practical applications

The results of the study could have a number of important practical implications. Namely, understanding the time-course of recovery can allow practitioners to make more informed decisions when devising the training schedule around competitive fixtures, providing valuable ancillary information when prescribing training in the 72 h following competitive match-play. In addition, understanding the magnitude of fatigue and the time-course of recovery can assist in decision making regarding player rotation strategies during congested fixture schedules, which are commonplace in modern day soccer. Furthermore, understanding the etiology of fatigue is critical when determining the potential efficacy of recovery interventions aimed at accelerating the natural time-course of recovery in an attempt to facilitate performance and reduce the likelihood of injury during subsequent activity. The results of the study could thus provide a scientific basis for research and practice concerning the implementation of recovery strategies following match-play. Finally, the lack of association and differential time-course of recovery between neuromuscular, physical function and perceptual measures in the present study suggests that practitioners should use a range of both subjective and objective measures when monitoring fatigue and recovery in order to provide a more comprehensive understanding of readiness to train/compete.

## Author contributions

CB, KT, and SG contributed to the conception/design of the work and contributed to the interpretation and analysis of the data. CB, KT, SG, PP, JD, and KH acquired the data for the study. All authors have drafted/revised the intellectual content and revised the final version. All listed authors qualify for authorship.

### Conflict of interest statement

The authors declare that the research was conducted in the absence of any commercial or financial relationships that could be construed as a potential conflict of interest.
