# Supplementary material for: Etiology and Recovery of Neuromuscular Fatigue following Competitive Soccer Match-Play
Source: Front Physiol. 2017 Oct 25;8:831. doi: 10.3389/fphys.2017.00831 (PMC5661001; doi:10.3389/fphys.2017.00831)
Supplement: Supplementary file 1 [file Presentation1.PPT]

## Slide 1
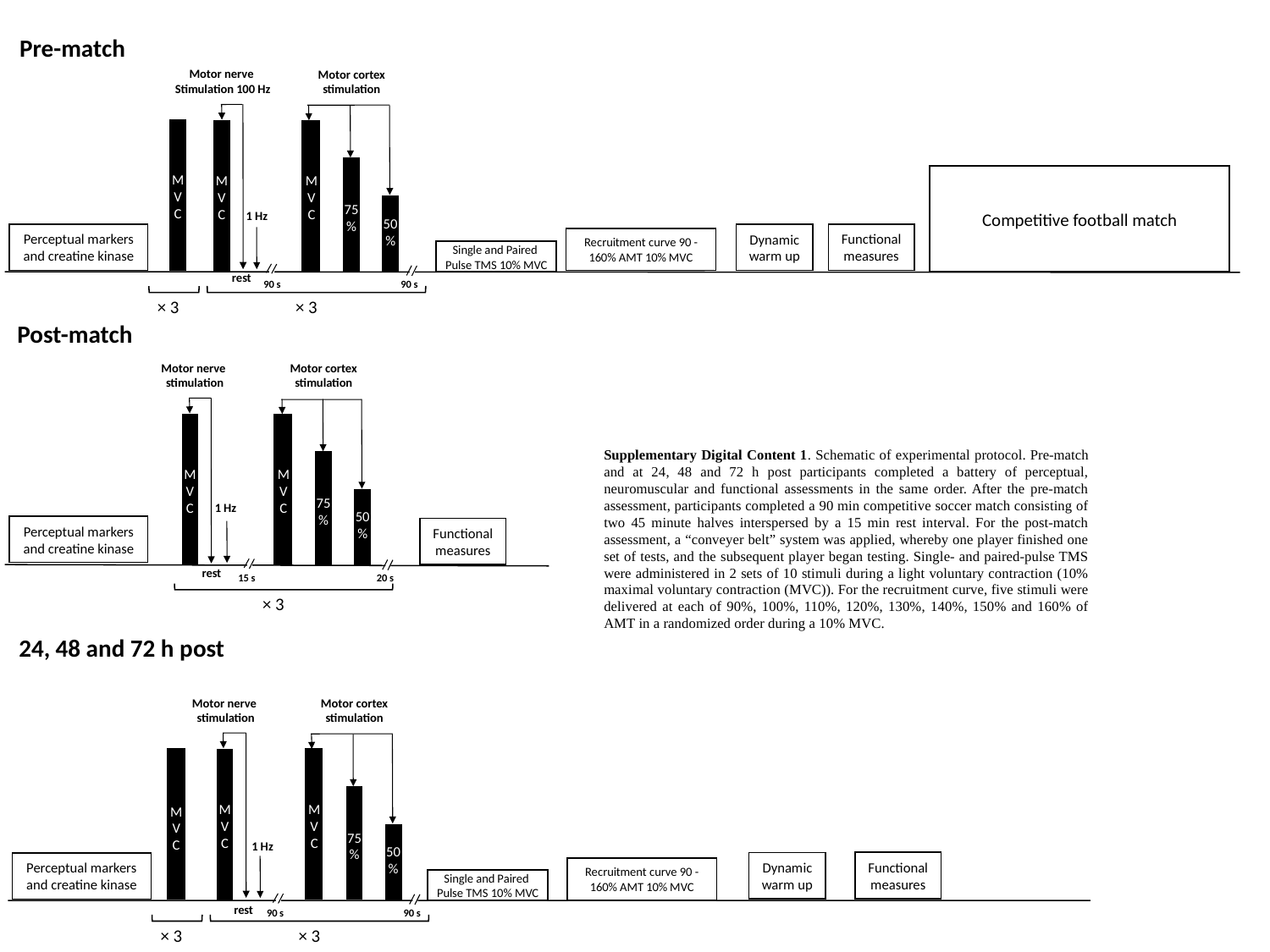

Pre-match
Motor nerve
Stimulation 100 Hz
Motor cortex
stimulation
M
V
C
75
%
50
%
M
V
C
M
V
C
M
V
C
M
V
C
M
V
C
75
%
50
%
M
V
C
50
%
M
V
C
1 Hz
Competitive football match
Perceptual markers and creatine kinase
Functional measures
Dynamic
warm up
Recruitment curve 90 -160% AMT 10% MVC
Single and Paired
Pulse TMS 10% MVC
90 s
rest
90 s
× 3
× 3
Post-match
Motor nerve
stimulation
Motor cortex
stimulation
M
V
C
75
%
50
%
M
V
C
50
%
15 s
20 s
rest
Supplementary Digital Content 1. Schematic of experimental protocol. Pre-match and at 24, 48 and 72 h post participants completed a battery of perceptual, neuromuscular and functional assessments in the same order. After the pre-match assessment, participants completed a 90 min competitive soccer match consisting of two 45 minute halves interspersed by a 15 min rest interval. For the post-match assessment, a “conveyer belt” system was applied, whereby one player finished one set of tests, and the subsequent player began testing. Single- and paired-pulse TMS were administered in 2 sets of 10 stimuli during a light voluntary contraction (10% maximal voluntary contraction (MVC)). For the recruitment curve, five stimuli were delivered at each of 90%, 100%, 110%, 120%, 130%, 140%, 150% and 160% of AMT in a randomized order during a 10% MVC.
1 Hz
Perceptual markers and creatine kinase
Functional measures
× 3
24, 48 and 72 h post
Motor nerve
stimulation
Motor cortex
stimulation
M
V
C
75
%
50
%
M
V
C
M
V
C
M
V
C
75
%
50
%
M
V
C
50
%
M
V
C
M
V
C
1 Hz
Functional measures
Dynamic
warm up
Perceptual markers and creatine kinase
Recruitment curve 90 -160% AMT 10% MVC
Single and Paired
Pulse TMS 10% MVC
90 s
rest
90 s
× 3
× 3
